# Laparoscopic cryoablation *vs.* percutaneous cryoablation for treatment of small renal masses: a systematic review and meta-analysis

**DOI:** 10.18632/oncotarget.15273

**Published:** 2017-02-10

**Authors:** Kehua Jiang, Kun Tang, Xiaolin Guo, Haoran Liu, Hongbo Chen, Zhiqiang Chen, Hua Xu, Zhangqun Ye

**Affiliations:** ^1^ Department of Urology, Institute of Urology, Tongji Hospital, Tongji Medical College, Huazhong University of Science and Technology, Wuhan, China; ^2^ Department of Urology, The Central Hospital of Enshi Tujia and Miao Autonomous Prefecture, Enshi, China

**Keywords:** Laparoscopic cryoablation, percutaneous cryoablation, small renal masses, meta-analysis

## Abstract

**CONTEXT:**

Laparoscopic cryoablation (LCA) and percutaneous cryoablation(PCA) have been used on patients with small renal masses(SRMs) for many years. However, clinical studies assessing their feasibility and safety have reported contradictory outcomes. This systematic evaluation was performed to obtain comprehensive evidence with regard to the feasibility and safety of PCA compared with LCA.

**METHODS:**

A systematic search of Embase, Pubmed, Medline, the Cochrane Library were performed to identify studies that compared LCA with PCA were published up to Mar 2016. Outcomes of interest included perioperative, pathologic variables, and complications.

**RESULTS:**

Thirteen studies estimating LCA *versus* PCA were included for meta-analysis. Patients undergoing PCA were significantly older(WMD = -0.16 years; *P* = 0.01) and patients with posterior tumors were significantly prefer undergoing PCA than LCA(OR = 0.23; *P* = 0.0007), whereas patients with anterior tumors were significantly prefer undergoing LCA(OR = 3.82; *P* = 0.02). although PCA was associated with shorter hospital stay(WMD = 1.17 days; *P* < 0.0001) and higher incidence rate of perirenal hematoma(OR = 0.18; *P* < 0.0001). All the other analyzed parameters were similar, regardless of the surgical approach.

**CONCLUSIONS:**

Patients undergoing PCA have shorter hospital stay and PCA was more frequently used in older patients and posterior tumors. Whereas LCA was associated with lower incidence rate of perirenal hematoma. Further multicenter, prospective and long-term follow-up RCTs are required to verify these findings.

## INTRODUCTION

Over the past decades, the morbidity of small renal masses(SRMs) has increasingly risen, with computed tomography (CT ) imaging is widely applied to various medical disciplines [1, 2]. The gold standard for the treatment of SRMs is open or laparoscopic partial nephrectomy(PN), and which shows excellent results, with 5-year survival rates approaching 97% [3, 4] However, PN is associated with intra- and post-operative complication rate of about 20% [3]. In the course of the past two decades, ablative techniques for instance, cryoablation have emerged as a less invasion treatment option in patients with significant comorbidities that may preclude extirpative surgery [4, 5]. Initially, cryoablation was applied to treat the patients declining surgical intervention or poor surgical elderly, thus became an alternative choice for SRMs and associated with better oncological outcomes compared with PN [4].

Cryoablation approaches are often performed laparoscopically under direct visualization or percutaneously under image-guided for SRMs. The advantages of laparoscopic cryoablation(LCA) is operation of probes with lower complication under direct visualization. Whereas, the advantages of the percutaneous cryoablation(PCA) are local anesthesia, less cost, shorter hospital stay, shorter recovery time and lower complication rates. In the last few years, several studies of comparing LCA with PCA applied to SRMs have reported perioperative outcomes [6–9]. which included cost, recovery time, hospital stay, procedure time, oncologic and functional outcomes. However, the published outcomes of LCA comparing with PCA have not been evaluated, and no definitive conclusions for reference to guiding their clinical application. Hence, we performed a systematic review of literature with a meta-analysis of the available published literature to compare LCA with PCA with respect to clinical characteristics, perioperative complications and oncological outcomes of SRMs patients.

## RESULTS

### Characteristics of eligible studies

**Figure 1:**
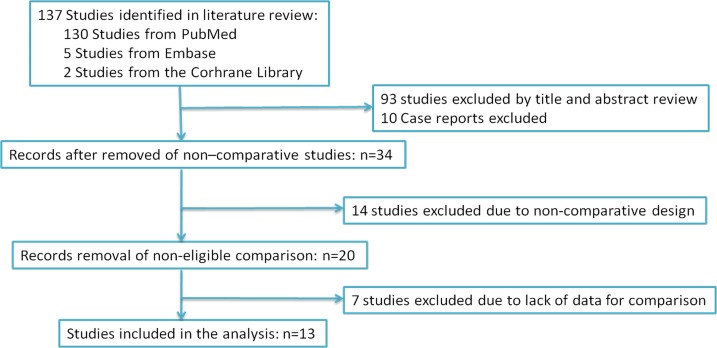
PRISMA diagram The search strategy and number of studies identified for inclusion in this meta-analysis.

According to search strategy, 13 studies [6–18] were included assessing LCA *vs*. PCA conformed to the inclusion criteria and were applied to performed this meta-analysis (Figure 1). The demographic and clinical characteristics of these literatures were shown in Table 1.

**Table 1:** Characteristics of included studies

First author year	Country	Study interval	Design	LOE	No.of patients LCA/PCA	Matching/ comparable*
Bandi, 2008	USA	2000-2006	Retrospective	3b	58/20	1, 2, 3, 5, 6, 7, 10, 12
Derweesh, 2008	USA	1997-2007	Retrospective	3b	34/26	1, 3, 4, 5, 6, 7, 8, 10, 12
Finley, 2008	USA	2003-2007	Retrospective	3b	19/18	2, 5, 7, 12
Goyal, 2012	USA	1997-2008	Retrospective	3b	53/141	1, 3, 4, 5, 7, 8, 9, 10, 12
Hinshaw, 2008	USA	2001-2007	Retrospective	3b	60/30	1, 3, 4, 7, 12
Kim, 2014	USA	2001-2011	Retrospective	3b	145/118	1, 2, 3, 4, 7, 8, 12
Malcolm, 2009	USA	2003-2007	Retrospective	3b	46/20	1, 2, 5, 7, 12
Mues, 2010	USA	2005-2008	Retrospective	3b	81/90	1, 4, 5, 7, 12
Rofriguez, 2015	Spain	2007-2013	Retrospective	3b	40/40	1, 2, 3, 4, 6, 7, 8, 9, 12
Strom, 2011	USA	1998-2010	Retrospective	3b	84/61	1, 2, 3, 6, 7, 8, 12
Trudeau, 2016	Canada	2000-2009	Retrospective	3b	289/227	1, 3, 4, 7, 9, 12
Tsivian, 2010	USA	2001-2008	Retrospective	3b	72/123	1, 2, 3, 5, 7, 8, 9, 12
Zargar, 2015	USA	1997-2012	Retrospective	3b	275/137	1, 2, 3, 4, 6, 7, 12

LCA= Laparoscopic cryoablation; PCA= Percutaneous cryoablation; LOE= level of evidence.

*:Matching/comparable variable: 1=age, 2=BMI, 3=gender, 4=laterality(right/left), 5=number of mass, 6=ASA score, 7=tumor size, 8=tumor location, 9=CCI(Charlson Comorbidity Index), 10=No of probes used per lesion, 11=cost, 12=follow up

### Quality of the studies and level of evidence(Table 1)

In this meta-analysis, the Newcastle-Ottawa Scale quality assessment method of the observational studies [19], and the US Preventive Services Task Force grading system [20] were applied to evaluate the quality of include studies. Also, the demographic variables of LCA and PCA were extracted independently from included literatures (Table 1).

### Description of included studies and patients Demographics(Table 2)

Patients undergoing LCA were significantly younger(WMD = -0.16 years; 95% CI: -0.29 to -0.04; *P* = 0.01)(Table 2) than PCA, patients with posterior tumors were significantly prefer undergoing PCA than LCA(OR = 0.23; 95% CI: 0.10 to 0.54; *P* = 0.0007) (Table 2). Whereas patients with anterior tumors were significantly prefer undergoing LCA(OR = 3.82; 95% CI: 1.21 to 12.07; *P* = 0.02)(Table 2) than PCA. There were no statistical differences in term of gender(OR = 0.89; 95% CI: 0.74 to 1.07; *P* = 0.22), body mass index(BMI)(WMD = -0.78kg/m^2^; 95% CI: -2.43 to 0.86; *P* = 0.35), tumor size (WMD = -0.07 cm; 95% CI: -0.28 to 0.15; *P* = 0.55), tumor polarity(upper pole: OR = 1.26; 95% CI: 0.94 to 1.67; *P* = 0.12; midpolar: OR = 1.07; 95% CI: 0.64 to 1.77; *P* = 0.180; lower pole: OR = 0.77; 95% CI: 0.44 to 1.37; *P* = 0.38), and preoperative creatinine(WMD = -0.00 mg/dl; 95% CI: -0.13 to 0.12; *P* = 0.96) (Table 2).

**Table 2:** Overall analysis of demographic and clinical characteristics compared LCA with PCA

Outcomes of interest	No. of studies	No. of patients LCA/PCA	OR/WMD(95% CI)^†^	*p*-value	Study heterogeneity			
					Chi2	df	*I*^2^	*p*-value
Age(year)	6	638/412	−0.16[−0.29,-0.04] †	**0.01**	5.43	5	8%	0.37
BMI(kg/m2)	5	578/382	−0.78[−2.43,0.86] †	0.35	11.65	4	66%	**0.02**
Proportion/male	10	1110/923	0.89[0.74,1.07]	0.22	15.02	9	40%	0.09
Tumor size(cm)	6	444//365	−0.07[−0.28,0.15] †	0.55	14.66	5	66%	0.01
**Tumor location** anterior posterior central lateral	6 6 4 2	436/538 458/543 251/380 221/276	3.82[1.21,12.07] 0.23[0.10,0.54] 4.02[0.69,23.48] 0.98[0.58,1.65]	**0.02** **0.0007** 0.12 0.93	60.02 33.05 19.64 0.68	5 5 3 1	92% 85% 85% 0%	**<0.001** **<0.001** **0.0002** 0.41
**Tumor polarity** Upper pole Midpolar Lower pole	6 6 6	458/543 458/543 458/543	1.26[0.94,1.67] 1.07[0.64,1.77] 0.77[0.44,1.37]	0.12 0.80 0.38	6.14 15.57 19.59	5 5 5	19% 68% 74%	0.29 **0.008** **0.001**
Preoperative creatinine(mg/dl)	2	115/116	−0.00[−0.13,0.12] †	0.96	0.00	1	0%	0.94

LCA= Laparoscopic cryoablation; PCA= Percutaneous cryoablation; OR = odds ratio; WMD = weighted mean difference; CI = confidence interval; BMI = body mass index; † :WMD

### Outcomes of perioperative variables(Table 3)

**Figure 2:**
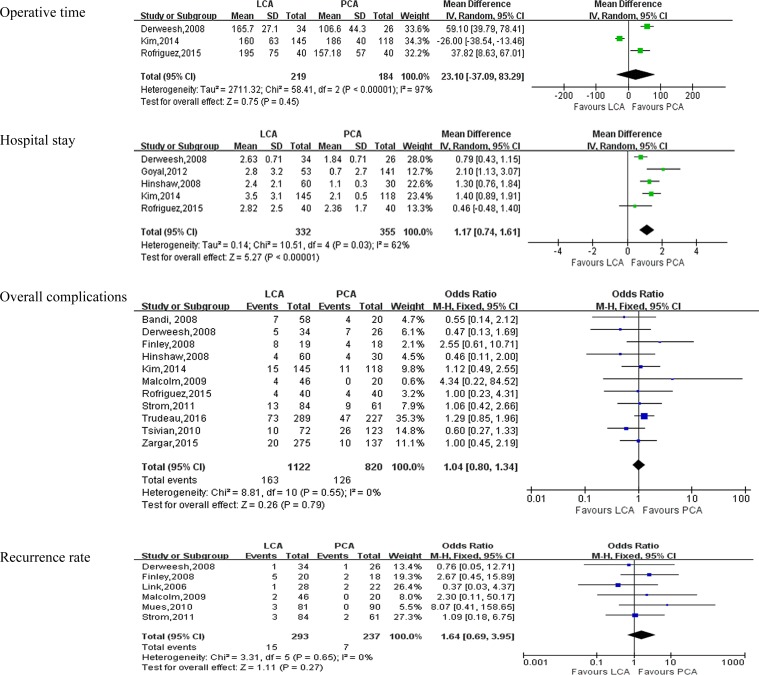
Forest plot and meta-analysis of postoperative outcomes comparing LCA with PCA LCA = laparoscopic cryoablation; PCA = percutaneous cryoablation.

With respect to perioperative variables, Pooling data of 5 studies [8, 9, 13–15] involving 687 participants found that PCA was associated with shorter hospital stay than PCA(WMD:1.17 days; 95% CI: 0.74 to 1.61; *P* < 0.0001)(Figure 2). However, there were no statistically difference between PCA and LCA in term of operative time(WMD = 23.10 minutes; 95% CI:-37.09 to 83.29; *P* = 0.45) (Figure 2), No of probes used per lesion(OR = -0.51; 95% CI:-1.49 to 0.47; *P* = 0.31)(Table 3, Supplementary Figure S1), transfusion rate(OR = 2.10; 95% CI: 0.79 to 5.59; *P* = 0.14)(Table 3, Supplementary Figure S1), postoperative creatinine (WMD = 0.11 mg/dl; 95% CI:-0.03 to 0.26; *P* = 0.12)(Table 3, Supplementary Figure S1).

**Table 3:** Overall analysis of perioperative outcomes comparing LCA with PCA

Outcome of interest	No. of studies	No.of patients LCA/PCA	OR/WMD(95%CI)^†^	*p*-value	Study heterogeneity			
					Chi2	df	*I*^2^	*p*-value
Operative time, min	3	219/184	23.10[−37.09,83.29] †	0.45	58.41	2	97%	**<0.0001**
No of probes used per lesion	2	87/167	−0.51[−1.49,0.47]	0.31	19.58	1	95%	**<0.0001**
Hospital stay,days	5	332/355	1.17[0.74,1.61] †	**<0.0001**	10.51	4	62%	**0.03**
Transfusion rate	5	265/215	2.10[0.79,5.59]	0.14	1.85	4	0%	0.76
Postoperative creatinine(mg/dl)	2	115/116	0.11[−0.03,0.26] †	0.12	1.64	1	39%	0.20

LCA= laparoscopic cryoablation; PCA= percutaneous cryoablation; OR = odds ratio; WMD = weighted mean difference; CI = confidence interval; † :WMD

### Outcomes of complications(Table 4)

**Figure 3:**
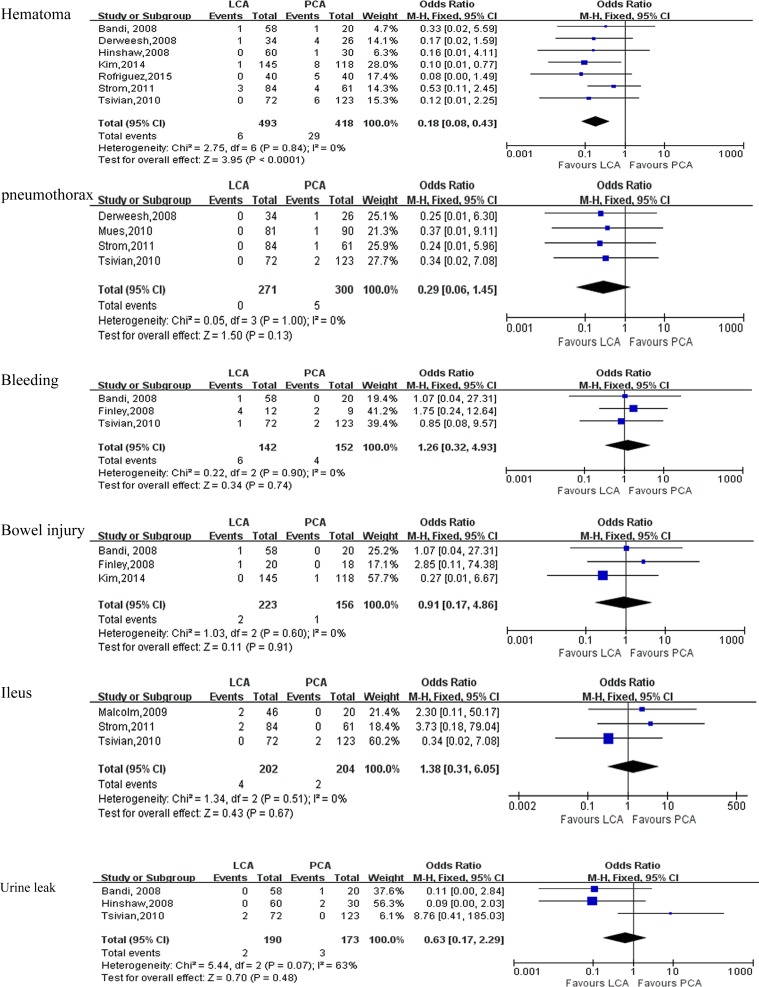
Forest plot and meta-analysis of complications between LCA and PCA LCA = laparoscopic cryoablation; PCA = percutaneous cryoablation.

Pooling data of 11studies [6, 7, 9, 10, 12–18] reported on perioperative complications. There was no statistical difference between LCA and PCA in term of overall complications(OR:1.04; 95% CI: 0.80 to 1.34; *P* = 0.79)(Figure 2). A meticulous classification of all perioperative complications showed that PCA had a higher incidence of perirenal hematoma (OR: 0.18; 95% CI: 0.08 to 0.43; *P* < 0.0001) than LCA(Figure 3), whereas there were no statistically significant between LCA and PCA in term of pneumothorax(OR: 0.29; 95% CI: 0.06 to 1.45; *P* = 0.13) (Figure 3), bleeding(OR:1.26; 95% CI: 0.32 to 4.93; *P* = 0.74) (Figure 3), bowel injury (OR:0.91; 95% CI: 0.17 to 4.86; *P* = 0.91) (Figure 3), ileus(OR:1.38; 95% CI: 0.31 to 6.05; *P* = 0.67) (Figure 3), urine leak(OR: 0.63; 95% CI: 0.17 to 2.29; *P* = 0.48)(Figure 3), artial fibrillation(OR: 2.45; 95% CI: 0.38 to 15.66; *P* = 0.34)(Table 4, Supplementary Figure S2), deep venous thrombosis(DVT) (OR:1.45;95% CI: 0.18 to 11.40;*P* = 0.73)(Table 4, Supplementary Figure S2), myocardial infarction(OR:1.59; 95% CI: 0.37 to 6.77;*P* = 0.53) (Table 4, Supplementary Figure S2), and neuropraxia(OR:0.28; 95% CI: 0.05 to 1.65; *P* = 0.16) (Table 4, Supplementary Figure S2).

**Table 4:** Overall analysis of complications comparing LCA and PCA

Outcome of interest	No. of studies	No.of patients LCA/PCA	OR (95%CI)	*p*-value	Study heterogeneity			
					Chi2	df	*I*^2^	*p*-value
Overall complications	11	1122/820	1.04 [0.80, 1.34]	0.79	8.81	10	0%	0.55
Artial fibrillation	3	275/261	2.45[0.38, 15.66]	0.34	0.46	2	0%	0.79
Bleeding	3	142/152	1.26[0.32, 4.93]	0.74	0.22	2	0%	0.90
Bowel injury	3	223/156	0.91[0.17, 4.86]	0.91	1.03	2	0%	0.60
DVT	2	165/136	1.45[0.18,11.40]	0.73	0.21	1	0%	0.64
Hematoma	7	493/418	0.18[0.08,0.43]	**<0.0001**	2.75	6	0%	0.84
Ileus	3	202/204	1.38[0.31,6.05]	0.67	1.34	2	0%	0.51
Myocardial infarction	4	573/468	1.59[0.37,6.77]	0.53	1.39	3	0%	0.71
Neuropraxia	2	118/50	0.28[0.05,1.65]	0.16	1.96	1	49%	0.16
Pneumothorax	4	271/300	0.29[0.06,1.45]	0.13	0.05	3	0%	1.00
Urine leak	3	190/173	0.63[0.17,2.29]	0.48	5.44	2	63%	0.07

LCA=laparoscopic cryoablation; PCA= percutaneous cryoablation; OR = odds ratio; WMD = weighted mean difference; CI = confidence interval; DVT=deep venous thrombosis.

### Outcomes of pathological and oncological variables(Table 5)

**Figure 4:**
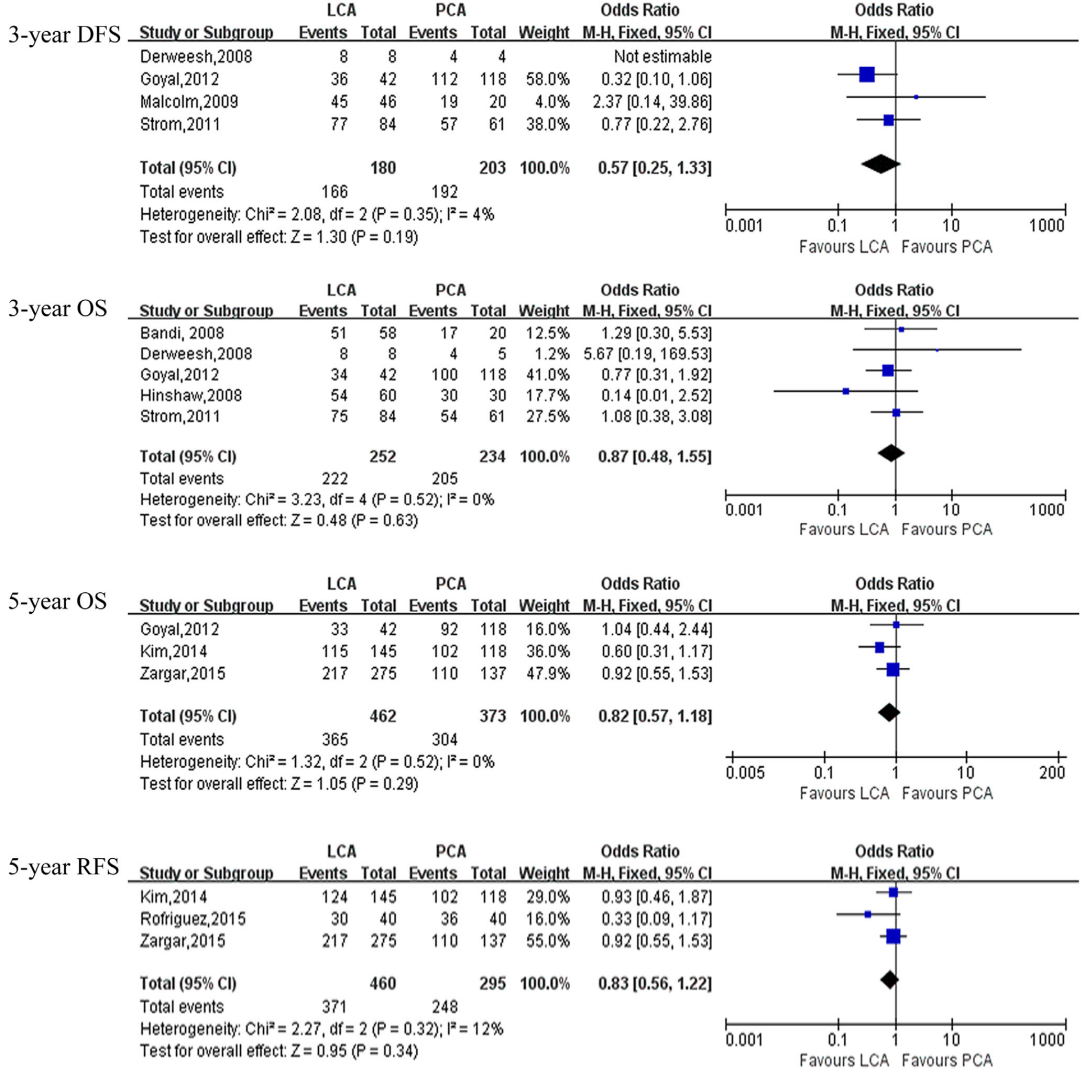
Forest plot and meta-analysis of oncological outcomes comparing LCA with PCA LCA = laparoscopic cryoablation; PCA = percutaneous cryoablation.

Pooling data of five [8, 9, 11, 13, 18] and seven [6, 8, 9, 11, 12, 14, 18] studies reported pathologic outcomes and local recurrence, respectively. The forest plot indicated that there was no statistical difference in term of postoperative pathologic outcomes (malignancy: OR: 1.21; 95% CI: 0.24 to 6.22; *P* = 0.82; benign: OR: 0.77; 95% CI: 0.16 to 3.74; *P* = 0.74)(Table 5, Supplementary Figure S3) and recurrence rate(OR: 0.95; 95% CI: 0.65 to 1.40; *P* = 0.81) (Figure 4). And there were also no statistical differences between PCA and LCA in term of 3-year disease-free survival(DFS)(OR: 0.57; 95% CI: 0.25 to 1.33; *P* = 0.19), 3-year overall survival(OS) (OR: 0.87; 95% CI: 0.48 to 1.55; *P* = 0.63), 5-year OS(OR: 0.82; 95% CI: 0.57 to 1.18; *P* = 0.29), 5-year recurrence-free survival(RFS)(OR: 0.83; 95% CI: 0.56 to 1.22; *P* = 0.34) (Figure 4, Table 5).

**Table 5:** Overall analysis of pathologic and oncological outcomes comparing LCA with PCA

Outcome of interest	No.of studies	No.of patients LCA/PCA	OR (95%CI)	*p*-value	Study heterogeneity			
					Chi2	df	*I*^2^	*p*-value
Pathologic								
Malignancy	5	591/367	1.21[0.24,6.22]	0.82	86.38	4	95%	<0.0001
Benign	5	591/367	0.77[0.16,3.74]	0.74	83.84	4	95%	<0.0001
Recurrence rate	7	756/597	0.95[0.65,1.40]	0.81	7.75	6	23%	0.26
3-year DFS	4	180/203	0.57[0.25,1.33]	0.19	2.08	2	4%	0.35
3-year OS	5	252/234	0.87[0.48,1.55]	0.63	3.23	4	0%	0.52
5-year OS	3	462/373	0.82[0.57,1.18]	0.29	1.32	2	0%	0.52
5-year RFS	3	460/295	0.83[0.56,1.22]	0.34	2.27	2	12%	0.32

LCA=laparoscopic cryoablation; PCA=percutaneous cryoablation; DFS=disease-free survival; OS=overall survival; RFS=recurrence-free survival; OR = odds ratio; WMD = weighted mean difference; CI = confidence interval.

## DISCUSSION

Laparoscopic PN approaches was associated with better surgical outcomes and had been recommend as the “golden standard” for SRMs. A large number of patients diagnosed with SRMs are aged people with comorbidities, hence, there is a high risk with these invasion surgical operations for these patients. Moreover, many of these patients carry competing risks which pose a greater mortality risk than do the SRM [21, 22]. Nowadays, cryoablation (CA) has attracted more interest for it *in situ* treatment tumor and less invasive. The cryoablation approaches offer several advantages than surgical excision, such as lower perioperative complications, shorter hospital stay, absence of renal ischemia, quicker time to recovery [22, 23]. As clinical outcome data of SRMs cryoablation with percutaneous and laparoscopic approaches begin to accumulate, The question arises as to which is preferable. Therefore, we conducted a meta-analysis to compared LCA with PCA and to evaluate its safety and feasibility.

Many surgeons general choose younger and good comorbidity condition patients to preform LCA. And our results showed that patients with older age and posterior tumor are more likely to undergo PCA. The reason of this differences was that the older and posterior tumor patients choose PCA to avoid injury of adjacent organs and decreased the intra- and post-operative complications. We also compared preoperative and postoperative creatinine level changes between the two approaches, and the results showed no significant difference.

Our study indicated that PCA provided a shorter hospital stay than LCA (WMD:1.17 days; *P* < 0.0001). The reason were that PCA to be performed on an outpatient basis, and avoidance of a general anesthetic can lead to significant saving in cost and time for patients and hospitals [24]. But our study results showed that there were no statistical differences between LCA and PCA in term of the other postoperative variables, such as operative time, No of probes used per lesion, and transfusion rate.

Hui et al [25] performed meta-analysis found that patients underwent surgically cryoablation had higher major complications than PCA, but our results showed that there was no statistical significant between LCA and PCA with respect to overall complications(OR:1.04; *P* = 0.79). And Kim et al [9] showed similar results and strengthens our results. This difference may be attributed to literature included in Hui's meta-analysis was not comparative studies and the sample was small. A subgroup analysis of overall complications indicated that PCA was associated with higher incidence rate of perirenal hematoma(OR: 0.18; *P* < 0.0001). The renal parenchymal fracture after LCA and PCA result in perirenal hematoma were the most common, and LCA was performed under the direction of visualization while PCA was guided by CT or ultrasound, This difference lead to PCA had higher incidence of perirenal hematoma than LCA. However, there were no significant differences between PCA and LCA in term of artial fibrillation, bleeding, bowel injury, DVT, ileus, neuropraxia, pneumothorax, urine leak. One issue is the grading of complications and parameters of complication were not always reported in a available standardized way in included literature, while another issue is that the sample of the included studies is small. More multicenter, large sample, long follow-up RCTs are needed to offer more details about complications and further verify those findings.

As for the oncologic outcomes, our data showed that there were no statistical differences in term of pathologic outcomes(malignancy: OR: 1.21; *P* = 0.82; benign: OR: 0.77; *P* = 0.74) and recurrence rate(OR: 0.95; *P* = 0.81) compared with LCA group. There were also no statistical differences in term of 3-year DFS(OR: 0.57; *P* = 0.19), 3-year OS(OR: 0.87; *P* = 0.63), 5-year OS(OR: 0.82; *P* = 0.29), 5-year RFS (OR: 0.83; *P* = 0.34) between the two groups. Goyal et al [8] demonstrated the OS, RFS and CSF were 85.12%, 95.56% and 98% for the PCA group at 3 years and 81.72%, 93.75% and 100% for the surgical cryoablation at 3 years, respectively. Strom et al [12] reported on 145 patients with 42.3 months of follow-up with a significant difference local recurrence in the PCA and LCA group(16.4% *vs* 5.9%); and the 3-year OS and DFS were 88.9%, 93.7% for the PCA group and 89.3%, 91.7% for the LCA group, respectively. Zargar et al [18] reported on 412 patients who underwent PCA and LCA; the 5-year OS and RFS were 82%, 80% for PCA group and 89%,79% for LCA group, respectively. Our data showed that there were no significant differences in OS and RFS between PCA and LCA. But there is exist selection bias in term of oncologic outcomes between the two groups, for example, patients with older and high risk comorbidities are prefer undergoing PCA. Additionally, The follow-up duration have an effect on the oncological outcomes of two approaches.

However, There were several limitations exist when analyzed and interpreting results in our meta-analysis. The major limitation is lack of well designed prospective, randomized control studies in our meta-analysis. Indeed, there was no RCTs in our included literatures. Secondly, there was existed heterogeneities of studies, especially in the comparing of the continuous data such as the length of hospital stay, operative time, and these parameters were influenced by the heterogeneities of patients’ conditions, surgeon's surgical skills and the sample size of studies.

Nevertheless, Our meta-analysis compared LCA with PCA for treatment of SRMs was performed with adequate studies available for analysis. We used all available variables from included studies, including demographic and clinical characteristics, operative time, overall complications and oncological outcomes, to compare LCA with PCA for SRMs and to assess the evidence of the included literature with strict criteria. Here, our meta-analysis maybe provide up to date conclusions for the advantages and disadvantages of two approaches for treatment of SMRs.

In conclusion, LCA and PCA have similar short-term outcomes for SRMs in selected patients. Patients undergoing PCA have shorter hospital stay and PCA was more frequently used in posterior tumors and older patients, whereas LCA was associated with lower incidence of perirenal hematoma.

## MATERIALS AND METHODS

### Literature search strategy

According to the Cochrane Handbook recommendations, a systematic review of published literature was performed [26]. No ethicissues get involved in this dissertation. A systematic dissertation was conducted using Medline, Embase, Pubmed, CNKI and all relevant studies has been identified by the Cochrane Library. The following key words were used: “comparative studies”, “laparoscopic cryoablation”, “percutaneous cryoablation”, “laparoscopic renal cryoablation”, “percutaneous renal cryoablation”, “cryoablation”, and “small renal masses”.

### Data extraction and outcomes of interest

Two of the authors(JKH and TK) extracted data from the selected studies including: author identification, country, publication years, study design, age, No. of patients, operative approaches that were mentioned previously, and results of intervention. All disagreements about eligibility were reached a consensus through authors discussion. Perioperative outcomes including operative time, overall complications, Length of hospital stay(LOS), and oncological outcomes were compared between the two methods from all the studies that were finally selected. Overall complications were graded on the basis of the Clavien-Dindo system [27].

### Inclusion criteria and exclusion criteria

Studies should satisfy the following requirements (1) to compare LCA with PCA (2) to display on outcome of two approaches (3) to document the surgery as LCA or PCA (4) to clearly document indications for cryoablation with SRMs. Studies will be excluded if (1) the study was not satisfied inclusion criteria or (2) the outcomes of literature were not mentioned or the parameters were impossible to analysis for either LCA or PCA from the published findings.

### Study quality assessment

In accordance with the criteria of Centre for Evidence-Based Medicine in Oxford, we evaluated the level of evidence(LOE) of included sixteen studies. The Jaded Score was applied to evaluated the methodological quality of RCTs [28]. The Newcastle-Ottawa Scale(NOS) was applied to assessed the methodological quality of non-RCTs observational studies [19]. Two authors(JKH and GXL) evaluated the quality of the studies and discrepancies were rechecked by the third reviewer(CHB) and consensus was achieved by discussion.

### Statistical analysis

All meta-analysis were conducted by Review Manger 5.3(Cochrane Collaboration, Oxford, UK). Continuous and dichotomous variables were calculated by weighted mean differences (WMDs) and odds ratios(ORs). All analysis results were reported with 95% confidence intervals(CIs). I^2^ test and chi-square-based Q test were applied to evaluated the quantity of heterogeneity, and when I^2^ > 50%, the evidence was considered to have substantial heterogeneity, the random- effects(RE) model would be applied, otherwise, the fixed effects(FE) model was applied. The presence of publication bias was evaluated by Egger's test and funnel plot. Sensitivity analysis were used to estimate the influence of studies with a high risk of bias on the overall effect.

## SUPPLEMENTARY MATERIALS FIGURES AND TABLES



## Abbreviations

LCA: laparoscopic cryoablation
PCA: percutaneous cryoablation
CA: cryoablation
SRMs: small renal masses
PN: Partial nephrectomy
LOE: The level of evidence
WMDs: weighted mean differences
ORs: odds ratios
CIs: confidence intervals
RE: random- effects
FE: fixed effects
DFS: disease-free survival
OS: overall survival
RFS: recurrence-free survival
