# Supplementary material for: Laparoscopic cryoablation vs. percutaneous cryoablation for treatment of small renal masses: a systematic review and meta-analysis
Source: Oncotarget. 2017 Feb 10;8(16):27635–44. doi: 10.18632/oncotarget.15273 (PMC5432364; doi:10.18632/oncotarget.15273)
Supplement: Supplementary file 1 [file oncotarget-08-27635-s001.pdf]

Laparoscopic cryoablation *vs.* percutaneous cryoablation for treatment of small renal masses: a systematic review and meta-analysis

Supplementary Material

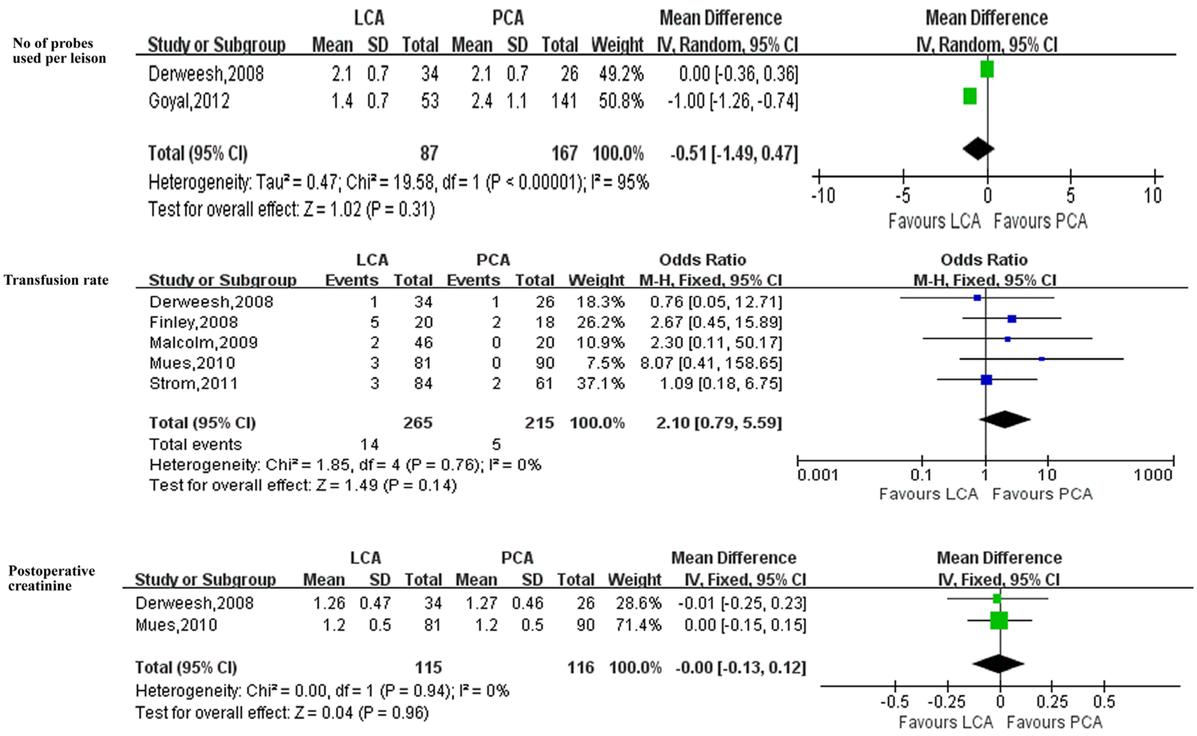

Supplementary Fig.S1: Forest plot and meta-analysis of postoperative outcomes between LCA and PCA. LCA=laparoscopic cryoablation; PCA= percutaneous cryoablation.

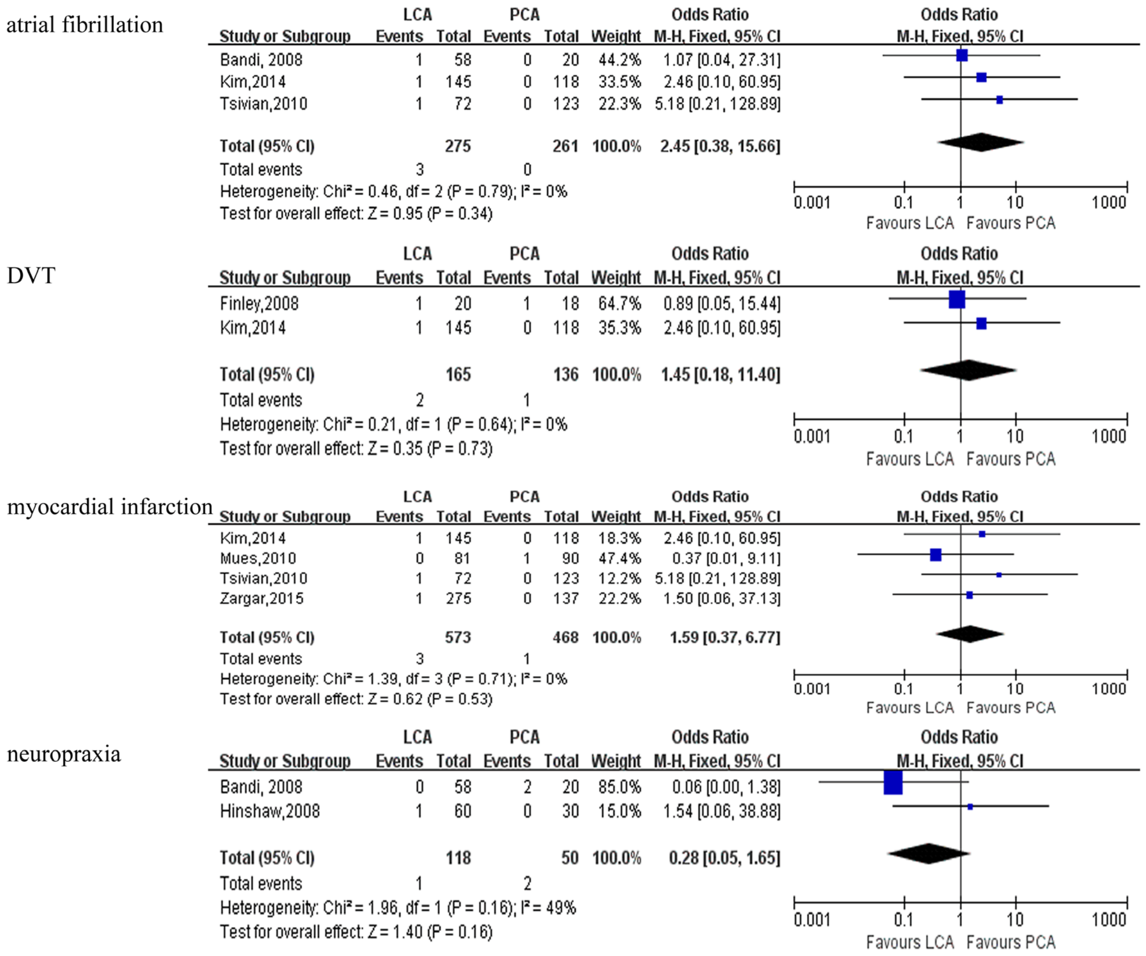

Supplementary Fig.S2: Forest plot and meta-analysis of complications between LCA and PCA. LCA=laparoscopic cryoablation; PCA= percutaneous cryoablation.

benign

Study or Subgroup	LCA Events	LCA Total	PCA Events	PCA Total	Weight	Odds Ratio M-H, Random, 95% CI
Derweesh,2008	5	29	3	21	17.8%	1.25 [0.26, 5.93]
Goyal,2012	23	53	71	141	20.8%	0.76 [0.40, 1.43]
Kim,2014	50	141	7	19	19.9%	0.94 [0.35, 2.54]
Mues,2010	39	98	9	67	20.4%	4.26 [1.89, 9.58]
Zargar,2015	52	275	102	137	21.1%	0.08 [0.05, 0.13]
**Total (95% CI)**		**596**		**385**	**100.0%**	**0.77 [0.16, 3.74]**
Total events	169		192			

Heterogeneity: $Tau^2 = 3.05$; $Chi^2 = 83.48$, df = 4 ($P < 0.00001$); $I^2 = 95\%$
Test for overall effect: Z = 0.33 (P = 0.74)

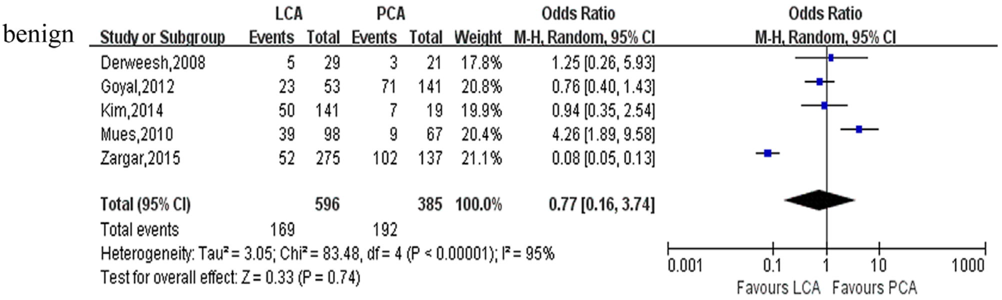

malignancy

Study or Subgroup	LCA Events	LCA Total	PCA Events	PCA Total	Weight	Odds Ratio M-H, Random, 95% CI
Derweesh,2008	24	29	18	21	18.0%	0.80 [0.17, 3.79]
Goyal,2012	25	48	52	123	20.7%	1.48 [0.76, 2.90]
Kim,2014	74	141	12	19	19.9%	0.64 [0.24, 1.73]
Mues,2010	59	98	58	67	20.4%	0.23 [0.10, 0.53]
Zargar,2015	223	275	35	137	21.1%	12.50 [7.67, 20.37]
**Total (95% CI)**		**591**		**367**	**100.0%**	**1.21 [0.24, 6.22]**
Total events	405		175			

Heterogeneity: $Tau^2 = 3.25$; $Chi^2 = 86.38$, df = 4 ($P < 0.00001$); $I^2 = 95\%$
Test for overall effect: Z = 0.23 (P = 0.82)

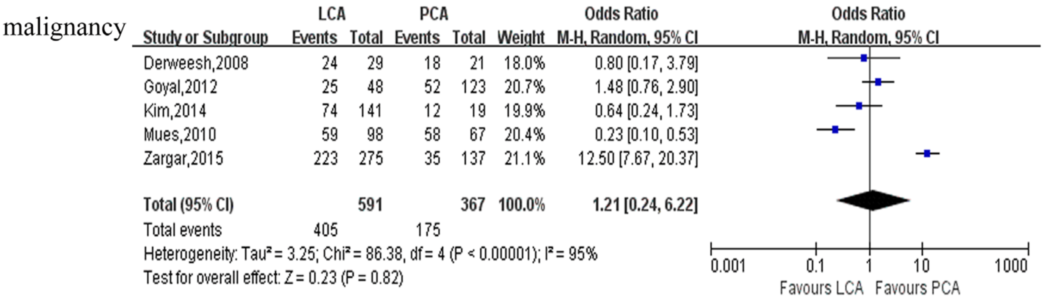

supplementary Fig.S3. Forest plot and meta-analysis of pathological outcomes between LCA and PCA. LCA=laparoscopic cryoablation; PCA= percutaneous cryoablation.
